# Lignin as a Binder Material for Eco-Friendly Li-Ion Batteries

**DOI:** 10.3390/ma9030127

**Published:** 2016-02-25

**Authors:** Huiran Lu, Ann Cornell, Fernando Alvarado, Mårten Behm, Simon Leijonmarck, Jiebing Li, Per Tomani, Göran Lindbergh

**Affiliations:** 1 Applied Electrochemistry, School of Chemical Science and Engineering, KTH Royal Institute of Technology, Stockholm SE-100 44, Sweden; niuniulu@kth.se (H.L.); behm@kth.se (M.B.); simonle@kth.se (S.L.); gnli@kth.se (G.L.); 2 Swerea SICOMP AB, Box 104, Mölndal SE-431 22, Sweden; 3 Innventia AB, Drottning Kristinas väg 61, Stockholm SE-114 28, Sweden; fernando.alvarado@innventia.com (F.A.); jiebing.li@innventia.com (J.L.); per.tomani@innventia.com (P.T.)

**Keywords:** lignin, binder, leaching, electrodes, Li-ion batteries

## Abstract

The industrial lignin used here is a byproduct from Kraft pulp mills, extracted from black liquor. Since lignin is inexpensive, abundant and renewable, its utilization has attracted more and more attention. In this work, lignin was used for the first time as binder material for LiFePO_4_ positive and graphite negative electrodes in Li-ion batteries. A procedure for pretreatment of lignin, where low-molecular fractions were removed by leaching, was necessary to obtain good battery performance. The lignin was analyzed for molecular mass distribution and thermal behavior prior to and after the pretreatment. Electrodes containing active material, conductive particles and lignin were cast on metal foils, acting as current collectors and characterized using scanning electron microscopy (SEM), electrochemical impedance spectroscopy (EIS) and galvanostatic charge-discharge cycles. Good reversible capacities were obtained, 148 mAh·g^−1^ for the positive electrode and 305 mAh·g^−1^ for the negative electrode. Fairly good rate capabilities were found for both the positive electrode with 117 mAh·g^−1^ and the negative electrode with 160 mAh·g^−1^ at 1C. Low ohmic resistance also indicated good binder functionality. The results show that lignin is a promising candidate as binder material for electrodes in eco-friendly Li-ion batteries.

## 1. Introduction

Li-ion batteries have attracted considerable attention due to their high energy density, high efficiency, long life and environmentally friendly operation. They have been widely used as power source for portable electronics, battery electric vehicles (BEVs) with zero emission, hybrid electric vehicles (HEVs) and plug-in hybrid electric vehicles (PHEVs) [1,2].

Typically, three components (active material, conductive material and a binder) are employed to assemble electrodes in Li-ion batteries. The conventional binder material for electrodes in commercial Li-ion batteries is poly vinylidene fluoride (PVDF), which binds strongly to the current collectors [3,4]. However, all fluorinated polymers can easily deteriorate the cycling performance of the batteries due to formation of stable LiF and double bonds (C=CF-) after reacting with Li metal or lithiated graphite (Li_x_C_6_) [5]. In addition, the reaction of PVDF and Li is exothermal, which can cause self-heating and thermal runaway [6]. Furthermore, PVDF needs to be dissolved in an organic solvent, typically *N*-methyl-2-pyrrolidinone (NMP), to produce slurry for the coating process. NMP is an expensive and toxic organic solvent, which is harmful to both humans and the environment. Therefore, it is essential to develop alternative binders that are non-fluorinated, less costly and more environmentally friendly. Recently, much effort has focused on using water-based binders instead of non-aqueous soluble binders, which could meet the requirements above. Water-based binders, such as styrene-butadiene-rubber (SBR), sodium-carboxyl-methyl-cellulose (CMC), poly (acrylamide-co-diallyldimethylammonium) (AMAC), and an SBR and CMC mixture binder have been widely evaluated as binder materials for electrodes in Li-ion batteries and they show similar bonding ability and high flexibility [7,8,9,10,11,12]. Magasinski *et al.* explored a novel binder consisting of poly (acrylic acid) (PAA) for Si-based anodes, which can be dissolved not only in water but also in an environmentally friendly organic solvent, ethanol [13]. Conductive materials, such as polyaniline (PANI), polypyrrole (PPy), and conducting polymer hydrogels (CPHs), have been demonstrated as binder materials for electrodes in Li-ion batteries, in particular where the active material undergoes volume changes during cycling [14,15,16]. However, more efforts should be undertaken to explore and search for binder materials for eco-friendly Li-ion batteries.

**Figure 1:**
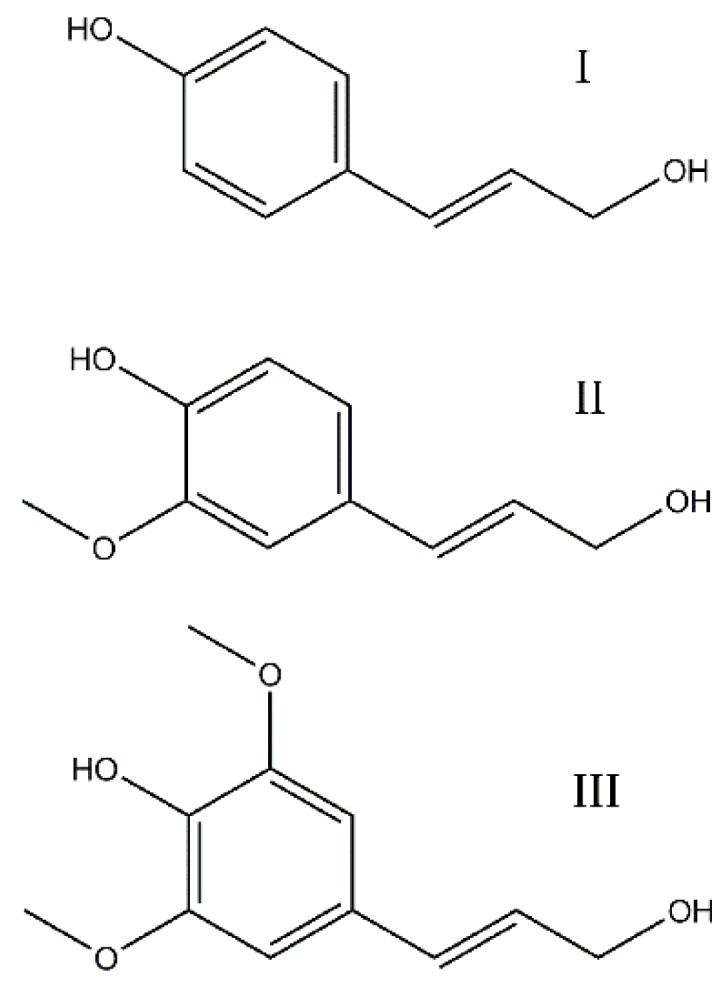
The primary precursors and building units of lignin: p-courmaryl alcohol (I); coniferyl alcohol (II); and sinapyl alcohol (III).

Lignin is a byproduct from the pulping industry that can be precipitated and separated from Kraft black liquors [17]. It is a macromolecule composed of phenylpropane units, built from themonomers p-coumaryl alcohol, coniferyl alcohol, and sinapyl alcohol (Figure 1) [18,19]. Recently, lignin has gained considerable attention for various applications because of its low cost, abundant and renewable nature. Bearing the energy crisis and environmental pollution in mind, lignin has been evaluated in applications such as fuel, fusing binder in briquetted anthracite fines, asphalt antioxidants and biofuels [20,21,22]. Milczarek *et al.* studied how lignin can be used to modify electrodes due to the formation of electroactive quinone functionalities, which can be oxidized and reduced during cycling [23,24]. He *et al*. suggested that a good electrochemical performance can be achieved using lignin-based composites to prepare carbon-based materials for Li-ion batteries [25]. Tenhaeff *et al.* found that lignin-based carbon fibers as free-standing anode material exhibit tunable electrochemical performance that can be used for both high-power and high-energy applications [26]. However, to the best of our knowledge, lignin is a material that has not directly been used as binder material for Li-ion batteries.

In this work, the possibility of using lignin as binder material for both LiFePO_4_ positive and graphite negative electrodes has for the first time been explored. Lignin was dissolved in acetone, which from an environmental perspective is a better solvent than NMP. Small fractions of lignin could dissolve in the electrolyte, which affected the performance of the electrodes. Pretreatment of lignin prior to preparing the electrodes was therefore necessary. Additionally, the effect of pressing the positive electrodes was studied, as this is a well-known process to decrease the porosity of the electrodes. In order to enhance the electrochemical performance of the negative electrodes, the effect of vinylene carbonate (VC) as an electrolyte additive was studied. The rate capabilities of both the positive and the negative electrodes are presented.

## 2. Experimental

### 2.1. Materials

Carbon-coated lithium iron phosphate (LiFePO_4_) of the type Life Power^®^ P2 was provided by Phostech Lithium. Graphite of the type Timrex SLP 30 AH-354 and Super-P-carbon were kindly obtained from Imerys Graphite & Carbon (Bironico, Switzerland) The densities of these materials were: LiFePO_4_—3.6 g/cm^3^, graphite—2.1 g/cm^3^, Super-P carbon—2.0 g/cm^3^, and lignin—about 1.5 g/cm^3^. Lignin prepared by means of the LignoBoost^®^ process was supplied by Innventia AB (Stockholm, Sweden). Diethyl carbonate (DEC) >99% purity for the pretreatment was purchased from Alfa Aesar (Karlsruhe, Germany). Polyethylene glycol (PEG) 400, dried acetone >99.9% purity and the electrolyte consisting of 1 M LiPF_6_ salt in ethylene carbonate (EC): DEC 1:1 by weight were obtained from Merck KGaA (Darmstadt, Germany). Vinylene carbonate (VC) as a 2 wt % additive to the electrolyte was purchased from Sigma-Aldrich Sweden AB (Stockholm, Sweden). Whatman filter paper as separator was purchased from VWR International (Stockholm, Sweden). The current collectors (aluminum foil with the thickness of 25 µm for positive electrodes and copper foil with the thickness of 20 µm for negative electrodes) were obtained from Advent Research Materials (Oxford, UK). All of the water used was deionized water.

### 2.2. Pretreatment of Lignin

In order to remove the small dissoluble fractions of the lignin and improve the stability of the lignin in the electrolyte, leaching was performed. The lignin was mixed in DEC by a magnetic stirrer at 500 rpm at least overnight. The suspension was then vacuum filtered through a Durapore membrane filter, type 2.22 µm GV, supplied by Merck Millipore (Billerica, MA, USA). The process was repeated several times until there was no coloring of the DEC. The resulting powder was dried at 60 °C for 12 h.

### 2.3. Preparation of LiFePO4 Positive and Graphite Negative Electrodes

Since lignin is a macromolecule, PEG can be used as a plasticizer to make the lignin less brittle as a film. Therefore, lignin was mixed with PEG to make the electrodes more elastic. Lignin, containing 5% PEG, was dissolved in acetone and then LiFePO_4_ or graphite, Super-P carbon and water (to a concentration of approximately 10%) were added. In order to optimize the capacity, different component ratios of electrodes were investigated. The lower the amount of inactive materials, such as carbon and binder, the higher the energy density. Meanwhile, the electrode should have good conductivity and mechanical integrity. The slurries were thoroughly mixed by magnetic stirrer at 500 rpm at least overnight. The electrodes were assembled by the following process: a current collector (25 µm Al foil for positive electrodes and 20 µm Cu foil for negative electrodes) was put on the top of an Elcometer 4340 Automatic film Applicator (Elecometer, Aalen, Germany). The slurry was spread on the current collectors by a doctor-blade with a 50 µm gap to produce the thin electrode layers. The coated foils were then dried at 110 °C for 24 h under vacuum to remove water properly. In order to know the flexibility of the electrode “pen tests” were carried out, where the electrodes were folded around a pen. No electrode cracks were found when examining them by the naked eye. Then, the electrodes were stored in a glove-box under argon atmosphere. In general, duplicate samples were prepared, showing good reproducibility in galvanostatic cycling tests.

An abbreviated notation is used to present the formulation of each composite electrode. To exemplify, 80-11-9 indicates that the electrode consists of 80 wt % LiFePO_4_, 11 wt % Super-P carbon and 9 wt % lignin and a negative electrode of 90-2-8 contained 90 wt % graphite, 2 wt % Super-P carbon and 8 wt % lignin.

Pressing was investigated as a method to decrease the porosity of the electrodes and increase the contact between the particles. The electrodes were pressed between two flat plates at 22.5 MPa, room temperature, using a laboratory press (Fontune Presses, Vlaardingen, The Netherlands).

As reference electrodes, a NMP slurry composed of 80 wt % LiFePO_4_, 11 wt % Super-P carbon and 9 wt % PVDF for positive electrode and a NMP slurring consisted of 90 wt % graphite, 2 wt % Super-P carbon and 8 wt % PVDF for negative electrode were prepared coating onto an aluminum and copper foil, respectively. Subsequently, NMP was evaporated in vacuum oven at 110 °C overnight. An abbreviated notation is used to present the formulation of each composite electrode.

### 2.4. Characterization

#### 2.4.1. Analysis of the Lignin and Morphology of the Electrodes

The molecular mass distribution (MMD) of the lignin with and without pretreatment was determined by size exclusion chromatography (SEC) using tetrahydrofuran (THF) as the mobile phase. The SEC system consists of three columns, Styragel HR1, Styragel HR2 and Styragel HR4, connected in series. The detection was performed using a refractive index detector (Waters 2414, Waters, Milford, MA, USA) and a UV detector (Knauer, Berlin, Germany). The UV absorbance was measured at 280 nm. The SEC system was calibrated using polystyrene standards. Prior to analysis, the extract, extracted and original lignin were derivatized by acetylation using acetic anhydride. The samples were dissolved in THF (approximately 5 mg/mL) and filtered (PTFE syringe filter, 0.20 µm, Toyo Roshi Kaisha, Ltd., Tokyo, Japan). Duplicate samples were analyzed. The molar mass distribution (MMD) was calculated from the RI signal.

In order to determine the decomposition temperature (T_d_) of the lignin with and without pretreatment and the eletrodes using lignin with and without PEG as binder, thermal gravimetric analysis (TGA) was performed by a TGA Q 5000 from TA Instruments. The lignins and electrodes were dried at 105 °C for 20 min before being heated at a rate of 15 °C/min up to 300 °C and 500 °C, respectively. Nitrogen was used as sample purge gas at a flow rate of 25 mL/min. The weight of the sample was registered throughout the heating and the decomposition temperature was read when the sample had lost 5% of its weight.

The glass transition temperature (T_g_) of the lignin with and without pretreatment was determined by differential scanning calorimetry (DSC) analysis. Between 1 and 4 mg samples were put in a DSC capsule and were dried for 2 hours at 105 °C. After cooling in a desiccator, the capsule was sealed airtight. The glass transition was determined with modulated DSC in a DSC Q 1000 from TA Instruments. A ramp from 20 °C up to 200 °C was performed with a rate of 3 °C/min. A sinusoidal modulation of the temperature of ±1 °C/60 s was superimposed on the temperature ramp to enhance the sensitivity. The T_g_ was determined from the midpoint of the reversing heat flow decrease. All report data are averages of duplicates.

Fourier Transform Infrared Spectroscopy (FTIR) spectra of lignin with and without pretreatment were recorded by a PerkinElmer Spectrum 2000 spectrometer (PerkinElmer Instrument, Waltham, MA, USA). The main measurement features were a spectral range from 4000 to 600 cm^−1^ with 16 scans at a resolution of 4 cm^−1^.

The morphologies of electrodes were examined using a Hitachi S-4800 (Hitachi, Tokyo, Japan) field emission Scanning Electron Microscopy (SEM).

#### 2.4.2. Electrochemical Evaluation

Pouch cells were built in a glove-box under an argon atmosphere. The composite electrodes containing LiFePO_4_ or graphite as active material were mounted *versus* Li metal as counter electrode, and the separator and electrolyte added. It contained VC at a concentration of 2 wt % when so indicated in the text. The electrochemical performance of the batteries was studied using a Gamry PCI4 G750 potentiostat (Gamry Instruments, Warminster, PA, USA). Cycling of the batteries was carried out at room temperature with the voltage limits of 2.8–4.0 V for the positive electrodes and 0.002–1.5 V for the negative electrodes. In order to investigate the effect of pressing, electrochemical impedance spectroscopy (EIS) was performed using a Gamry PCI4 G750 potentiostat between 0.01 Hz and 100 kHz. The cell was first charged at C/10 up to 10% state of charge (SOC).

### 2.5. Geometry

Electrode thickness was measured using a micrometer (Mitutoyo America Corporation, Aurora, IL, USA) with a resolution of 1 µm. The porosity (ε) of the LiFePO_4_ electrode based on lignin as binder material was estimated using the measured volume (V_1_) and the theoretical volume of the electrode (V_2_).

(1) $$\varepsilon =1-\frac{V_{2}}{V_{1}},$$

(2) $$V_{2}=r^{2}\times \pi \times t,$$

(3) $$V_{1}=\frac{m_{LFP}}{\rho_{LFP}}+\frac{m_{c}}{\rho_{c}}+\frac{m_{lignin}}{\rho_{lignin}},$$

where t is the electrode thickness; r is the radius of the electrode; $\rho_{LFP}$, $\rho_{c}$ and $\rho_{lignin}$ are LiFePO_4_, Super-P carbon and lignin densities given above, respectively; and $m_{LFP}$, $m_{c}$ and $m_{lignin}$ are the weights of LiFePO_4_, Super-P carbon and lignin in the electrode, respectively.

For the positive electrodes the thickness was approximately 14 µm, which is thin compared to commercial electrodes of about 35 µm. The reason is that the thicker the lignin based electrode, the more brittle was the composite electrode in this work. Therefore, in the future effort should be put on increasing the thickness of the electrode, possibly by modification of the lignin. The loading based on the mass of LiFePO_4_ varied from 1.3 to 1.5 mg·cm^−2^ with a porosity of about 60%. For the negative electrodes, the thickness and the loading of the graphite were about 27 µm and 1.3–1.4 mg·cm^−2^, respectively. The porosity was about 72%.

## 3. Results and Discussion

### 3.1. Pretreatment of Lignin

**Figure 2:**
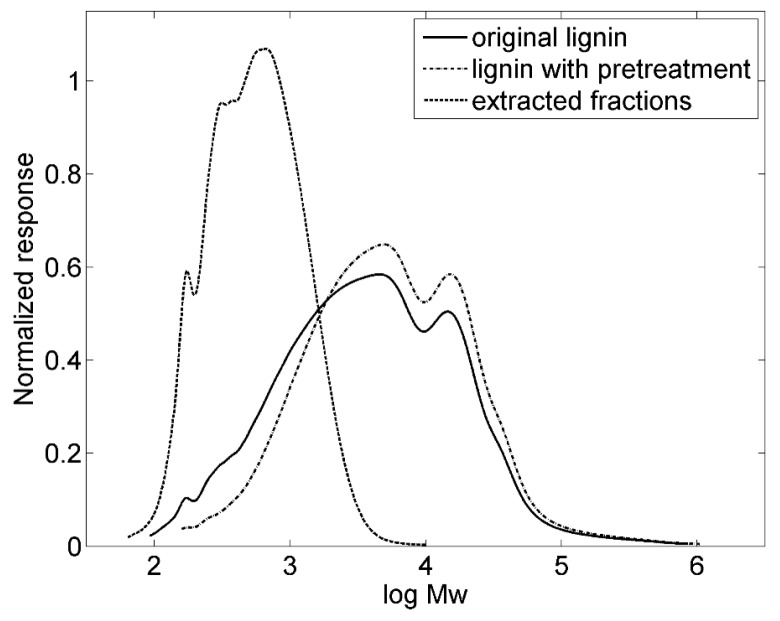
The molecular weight distribution for the three samples as measured using size exclusion chromatography.

Pretreatment of lignin was performed by the procedure described above, resulting in a concentration of lignin in the DEC phase of about 7 wt %. The original lignin, the pretreated lignin and the fractions extracted in DEC were analyzed in duplicate by SEC after acetylation, monitored simultaneously by RI and UV detectors. After calibration and normalization, highly repeatable molecular mass distributions (MMD) could be noticed for all the three samples. As illustrated in Figure 2, the shape of the MMD of the original and the pretreated lignin, respectively, are rather similar although the pretreated lignin shows a slightly more narrow distribution profile. The extracted fractions distributes in small molecular mass range. Obviously, the pretreatment procedure has been able to remove the small dissoluble fractions from the original lignin. Calculated MMD of the original lignin, pretreated lignin and extracted fractions are shown in Table 1. The pretreatment increased both the weight average molar mass (Mw) and the number average molar mass (Mn) of the lignin from 14,000 to 16,000 and 1400 to 2400, respectively, while the polydipersity (PD) index (Mw/Mn) was decreased from 9.9 to 6.8. The latter means that the pretreatment makes the distribution of molecular mass of the lignin more uniform.

**Figure 3:**
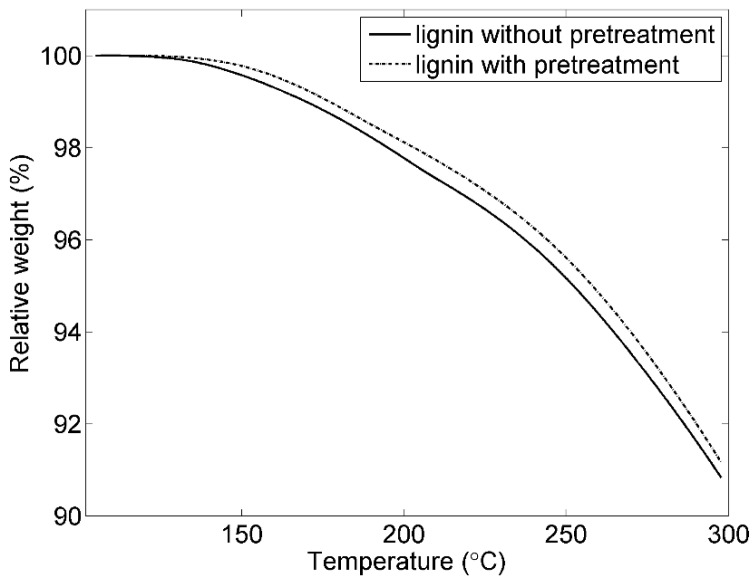
TGA thermograms of the lignin with and without pretreatment.

Thermal gravimetric analysis (TGA) was utilized to characterize the thermal behavior of the lignin with and without pretreatment. As shown in the Figure 3, their thermal behaviors are overall similar. The decomposition temperatures (T_d_) of the lignin with and without pretreatment are 255 and 257 °C, respectively, which means that both lignins are stable below these temperatures. Both T_d_ and volatiles (V) at 250 °C for the lignin with pretreatment are slightly higher than those of the original lignin (Table 2).

**Figure 4:**
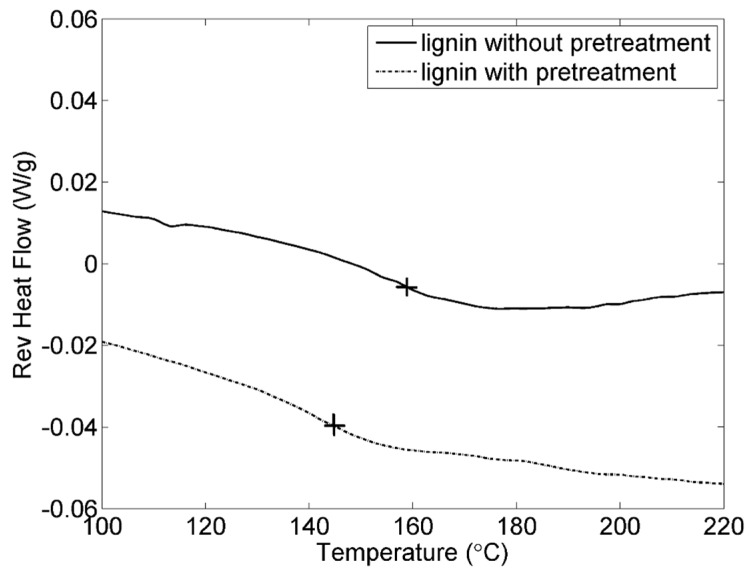
DSC of the lignin with and without pretreatment. The crosses show T_g_.

The reversing heat flow *versus* temperature is shown in Figure 4. It is seen that the glass transition temperature (T_g_) of the lignin with pretreatment is lower than the lignin without pretreatment. Table 2 shows that the averages T_g_ of the lignin with and without pretreatment are about 146 and 156 °C, respectively, where the lignin are converted into a rubber-like state. The lower of T_g_ for the lignin with pretreatment, the better chain flexibility, which could make the lignin as binder to be more elastic. It illustrates that pretreatment process could improve the physical properties for the lignin.

**Figure 5:**
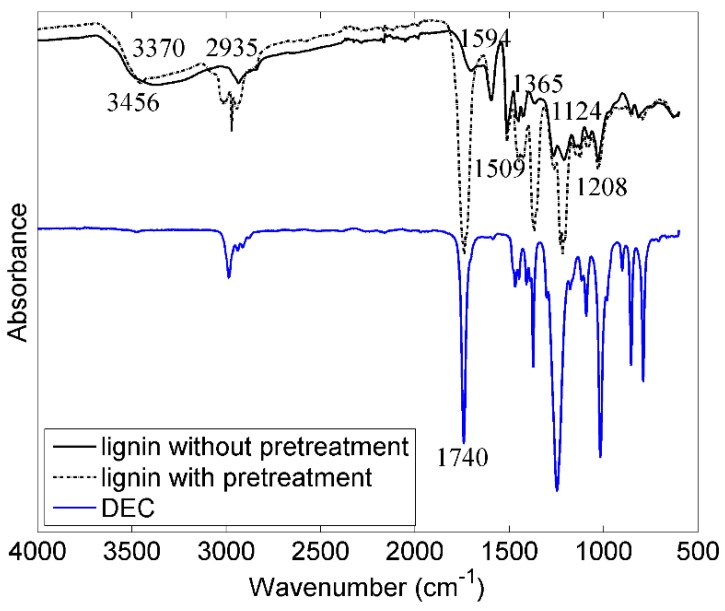
FTIR spectrum of DEC and of lignin with and without pretreatment. The lignin spectra were normalized based on the peak at wavenumber 1594 cm^−1^.

Figure 5 shows the FTIR spectra of lignin with and without pretreatment. Both lignins show a wide band between 3500 and 3100 cm^−1^ assigned to OH stretching vibrations from alcoholic and phenolic hydroxyl groups. Absorption bands at 2940–2820 cm^−1^ are assigned to O-H stretching in methyl and methylene groups. No absorption bands can be seen between 2800 and 1800 cm^−1^. Absorption bands located at 1740 cm^−1^ for the lignin with pretreatment are assigned to C=O stretch of DEC remaining in the lignin after pretreatment. However, DEC is one of the components of the electrolyte, which will not affect the performances in Li-ion batteries as shown in the following electrochemical data. For both lignin, the following bands could be found located at quite similar wavenumber: absorption bands at 1594 and 1509 cm^−1^ and 1450–1420 cm^−1^ are caused by aromatic ring vibrations, bands at 1365 cm^−1^ relate to C-H deformations bands at 1124 and 1028 cm^−1^ relate to methoxyl groups and those at 900–700 cm^−1^ relate to deformation vibrations of C-H-bonds on the benzene ring [18,27,28].

The chemical groups for both lignins are similar which illustrates that the lignin structure had not been changed by the pretreatment, however the overall intensity of peaks in FTIR for the lignin with pretreatment is higher, which indicates that pretreated lignin has a higher concentration of chemical groups after the pretreatment.

**Figure 6:**
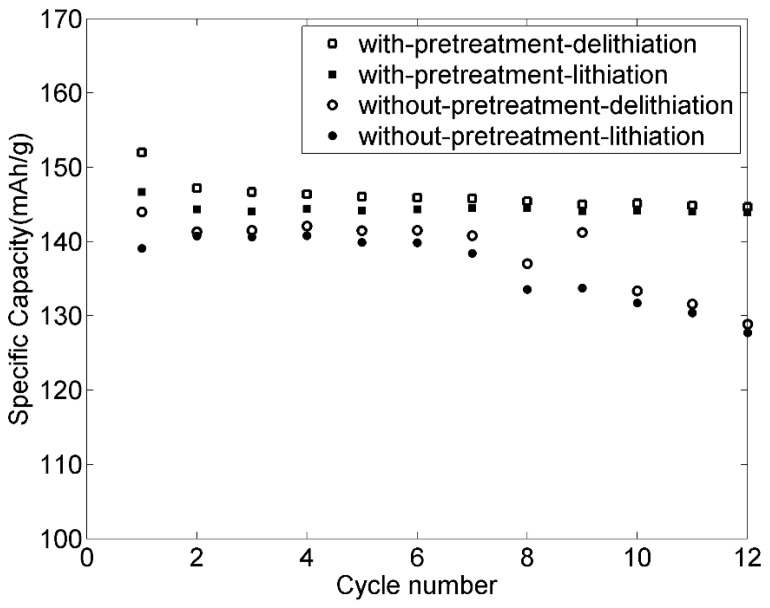
Specific capacity *versus* cycle numbers for 82-9-9 LiFePO_4_ positive electrodes with and without pretreatment cycled at C/10.

Specific capacities for the positive electrodes with and without pretreatment cycled at room temperature are shown in Figure 6. It can be seen that a specific capacity of about 140 mAh·g^−1^ was obtained for the pretreated electrode, which can be compared to the theoretical value of 170 mAh·g^−1^. The electrode functioned stably during repeated charge-discharge cycles without obvious reduction in performance. For the electrodes containing lignin that had not been pretreated by leaching, the specific capacity dramatically decreased after six cycles, as shown in Figure 6. Therefore, pretreatment is essential for making an electrode with lignin as binder, as this enhances the stability of the electrode.

### 3.2. The Effect of PEG for the Thermal Behaviours of the Electrodes

**Figure 7:**
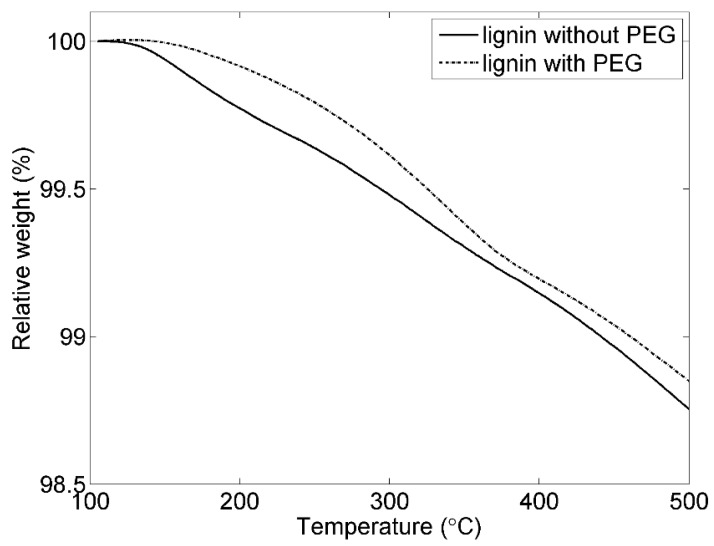
TGA thermograms of the 80-11-9 electrodes using lignin with and without PEG.

In order to investigate the effect of PEG as plastizer, TGA was utilized to characterize the results relating to the positive electrodes bound using lignin with and without PEG are shown in Figure 7. It is obvious that the decomposition temperatures (Td) of the electrode using lignin with PEG are higher than that of only lignin. The electrode without PEG started to lose weight at 125 °C. However, the electrode with PEG began to decompose at 151 °C. Volatiles (V) at 250 °C for the electrode with PEG was about 99.8%, which is higher than 99.6% without PEG.

### 3.3. Morphology of the Electrodes

**Figure 8:**
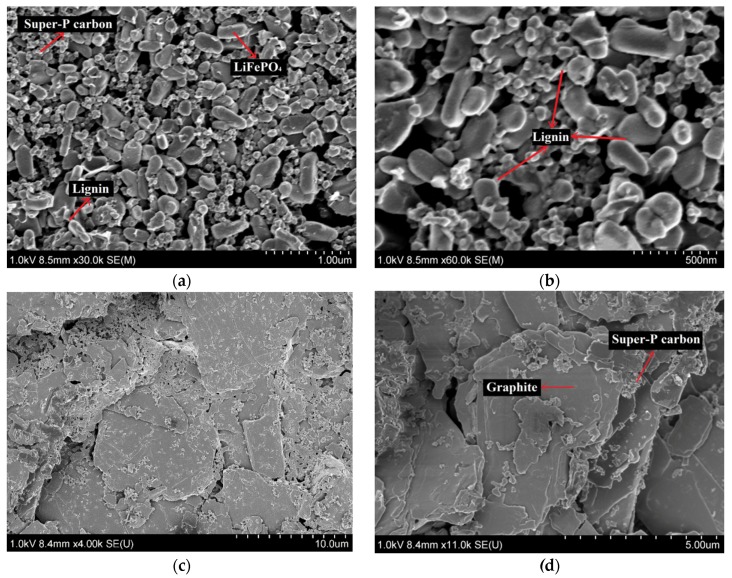
SEM images of the electrodes prepared using pre-treated lignin as binder material at different magnifications: (**a**,**b**) 82-9-9 LiFePO_4_ positive electrode; and (**c**,**d**) 90-2-8 graphite negative electrode.

Figure 8 shows SEM photos of the electrode surfaces. As shown in Figure 8a, the particles of the positive electrode are homogeneously dispersed. The large particles are LiFePO_4_ and the small particles are Super-P carbon. Lignin acts as a bridge to connect and bind the different particles together as shown in Figure 8b. In Figure 8c,d, it can be seen that the graphite flakes are relatively uniformly dispersed with Super-P carbon particles.

### 3.4. The Effect of Pressing for the Positive Electrodes

**Figure 9:**
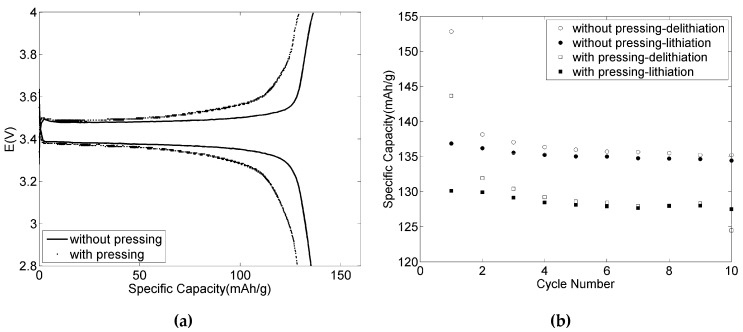
Galvanostatic 4th charge/discharge voltage profiles cycled at C/10 at room temperature (**a**) and specific capacity *versus* cycle numbers (**b**) for 82-9-9 LiFePO_4_ positive electrodes with and without pressing.

The porosity of the LiFePO_4_ positive electrodes without pressing was about 60% assuming $\rho_{lignin}$ to be 1.5 g·cm^−3^. Compared to the common commercial positive electrodes (with a porosity of about 30%), this is relatively high. After pressing as described above, the porosity of the electrodes slightly decreased to 55%. The electrochemical performance of the 82-9-9 LiFePO_4_ positive electrodes with and without pressing was tested at C/10 rate at room temperature and the results are shown in Figure 9. The figure clearly shows differences both in the voltage profile and the specific capacity before and after pressing. A high polarization and a significant decrease of specific capacity from 135 to 125 mAh·g^−1^ after 10 cycles were observed after pressing. This illustrates that pressing is not feasible for the electrodes using this type of lignin as binder. Pressing may be required with modified lignin or a thicker electrode.

**Figure 10:**
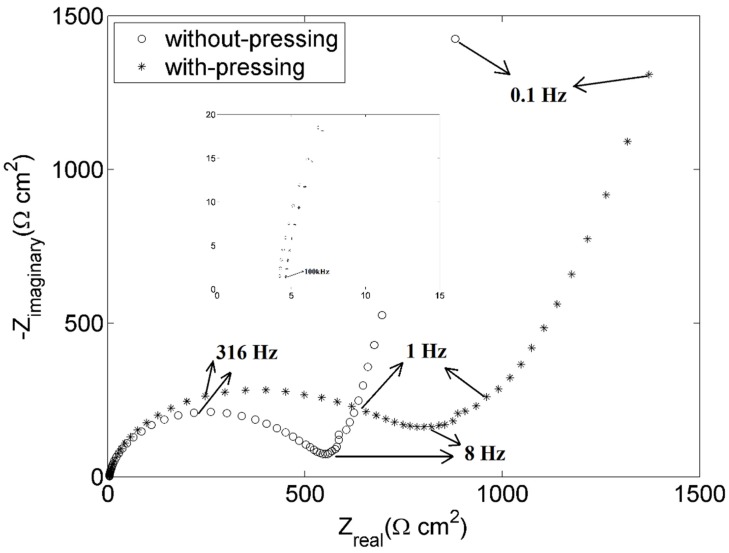
Nyquist plots of 82-9-9 LiFePO_4_ positive electrodes with and without pressing charged to 10% SOC at room temperature.

Figure 10 shows the Nyquist plots of the electrodes with and without pressing. The inset of Figure 10 shows that the ohmic resistances for both electrodes are quite similar, and determined to be about 4.2 Ω·cm^2^ and 4.6 Ω·cm^2^ for the electrode without and with pressing, respectively. These low values of resistance indicate that both electrodes have good contact with the current collector and that lignin as binder has good adhesion properties. The semi-circle in the medium-frequency (half circle peaking at 316 Hz) shows the charge transfer resistance and the linear line at low frequency (between 8 and 0.1 Hz) is assigned to diffusion resistance of the electrodes. As shown in Figure 10, the charge transfer resistance of the electrode with pressing was higher than that without pressing, which may be ascribed to pressing the electrode reduces the surface area of the electrode material since some active and conductive particles may be covered by lignin. It caused higher polarization and lower specific capacity, as shown in Figure 9, and therefore further electrodes in this study were not pressed.

### 3.5. Variation of Component Ratios for the Positive Electrodes

**Figure 11:**
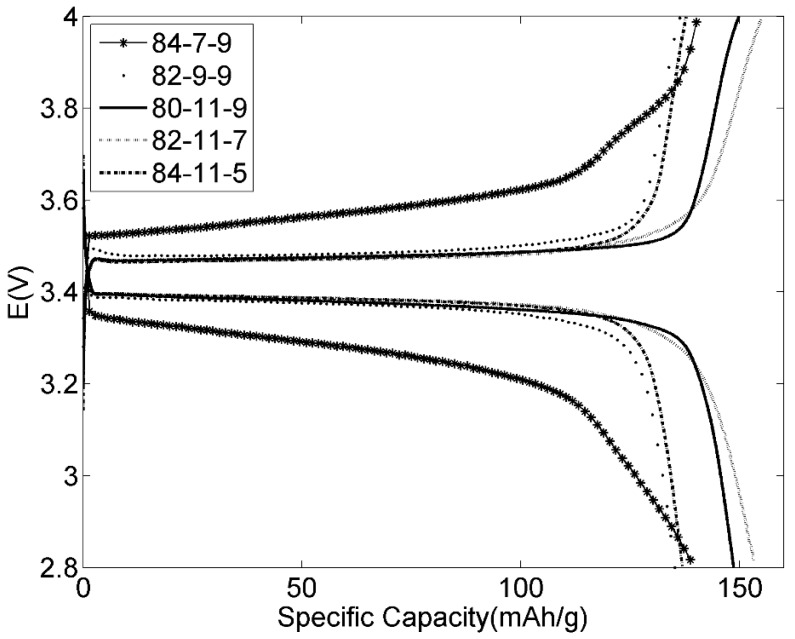
The 4th cycle of LiFePO_4_ positive electrodes *versus* Li metal with lignin binder at C/10 at room temperature.

For a high charge density the electrode should contain as little as possible of inactive materials, however addition of conductive particles (here Super-P carbon) is necessary for a high conductivity and a binder (here lignin) is necessary for the mechanical integrity. The amounts of Super-P carbon and lignin used in the electrode influence the electrochemical performance of Li-ion batteries [29] and it is necessary to optimize the composition of the electrodes. Figure 11 shows the voltage profiles for 84-7-9, 82-9-9, 80-11-9, 82-11-7 and 84-11-5 LiFePO_4_ positive electrodes with different percent of Super-P carbon and lignin. The 84-7-9 electrode with the lowest amount of Super-P carbon shows higher polarization than the other electrodes, which could be ascribed to poor electrical conductivity. Increasing the amount of Super-P carbon from 7 to 11 wt % while keeping the ratio of binder constant (84-7-9, 82-9-9 and 80-11-9) had a positive effect on the polarization and on the specific capacity. Increasing the binder concentration from 5 to 7 wt %, while keeping the ratio of conductive particles constant (84-11-5 and 82-11-7), clearly had a positive effect on the performance, while a further increase to 9 wt % (80-11-9) had no major effect. It was concluded from these results that the electrode should contain at least 11 wt % of Super-P carbon and at least 7 wt % lignin. Further experiments were made on the 80-11-9 electrode.

### 3.6. Electrochemical Performance for the 80-11-9 Positive Electrode

**Figure 12:**
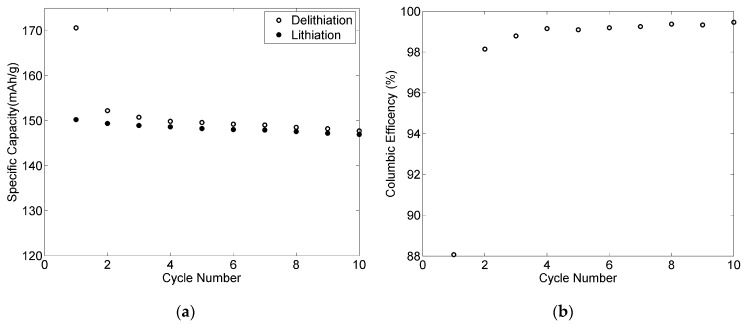
Electrochemical properties of the 80-11-9 positive electrode cycled at C/10: specific capacity (**a**) and coulombic efficiency (**b**) *versus* cycle number.

Figure 12 shows the specific capacity and coulombic efficiency during the first ten cycles of a LiFePO_4_ positive electrode. The first cycle coulombic efficiency (ratio discharge/charge) was 88%, after three cycles over 99% and then remained stable in subsequent cycles. High reversible capacity was obtained (about 148 mAh·g^−1^), which is not far away from the theoretical capacity of lithium iron phosphate using PVDF as a binder (approximately 170 mAh·g^−1^). The specific capacity was maintained throughout the subsequent cycles, indicating fairly good cycling performance.

### 3.7. Voltage Profile of the Graphite Negative Electrodes

**Figure 13:**
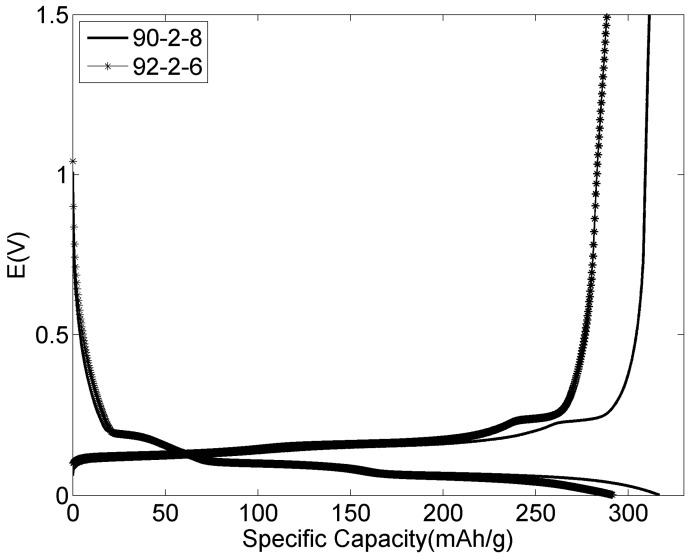
Galvanostatic charge/discharge voltage profiles of two graphite negative electrodes at C/10 rate during 4th cycle.

Lignin was also investigated as a binder material for the negative electrodes. The 4th charge/discharge voltage profiles of two graphite negative electrodes cycled at room temperature with 6 and 8 wt % lignin, respectively, are shown in Figure 13. The graph overall shows that lithium can intercalate and deintercalate into graphite with several distinct voltage plateaus according to different stages of lithium over one cycle, unaffected by the lignin. Comparing the 90-2-8 electrode and the 92-2-6 electrode, higher specific capacity and lower polarization were obtained for the former. It may be attributable to better binding properties for the 90-2-8 electrode and indicates that the electrode should contain more than 6 wt % lignin.

### 3.8. Effect of VC for the Graphite Negative Electrodes

**Figure 14:**
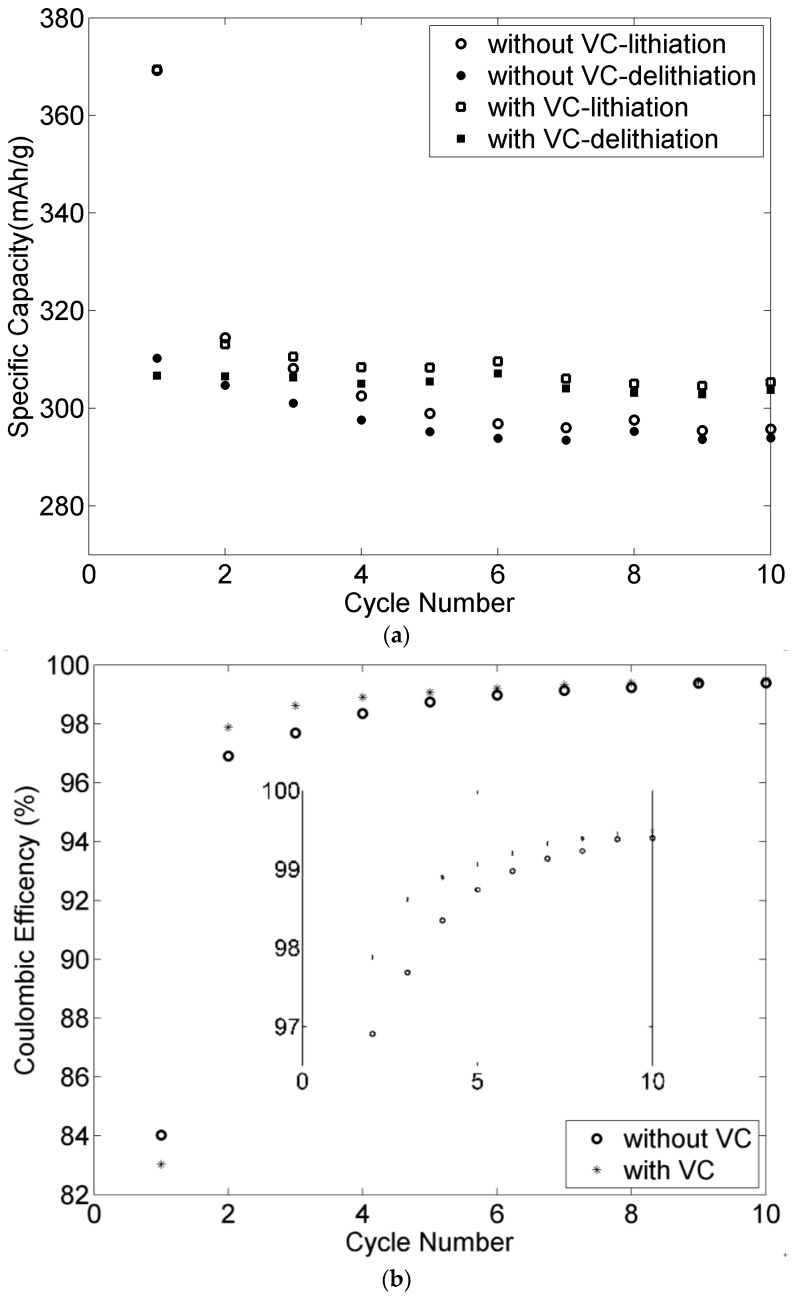
VC effect on the 90-2-8 graphite negative electrode using lignin as binder cycled at C/10 at room temperature. (**a**) Specific capacity; (**b**) Coulombic efficiency.

VC is a well-known additive for enhancing the cycling performance of an electrode [30]. The effect of VC for the negative electrode with lignin binder is presented in Figure 14. The observed irreversible capacity of the first cycle is attributed to formation of the solid electrolyte interface (SEI). After the first cycle, both the specific capacity and coulombic efficiency were overall slightly higher in the presence of VC as shown in Figure 14a,b. The specific capacity of the electrode with VC maintained a stable value of about 305 mAh·g^−1^ throughout the subsequent cycles. However, it decreased gradually for the electrode in VC-free electrolyte. The coulombic efficiency shown in Figure 14b was higher in the presence of VC during the first cycles, but the effect declined with prolonged cycling and had almost vanished after 10 cycles.

### 3.9. Rate Capabilities and Cycling Stability of the Electrodes

**Figure 15:**
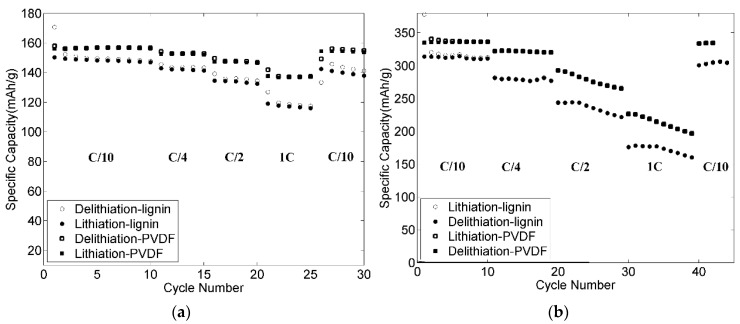
Rate capabilities for the electrodes *versus* a Li metal electrode: (**a**) 80-11-9 positive electrode; and (**b**) 90-2-8 negative electrode cycled with 2 wt % VC.

Rate capability evaluations of both positive and negative electrodes were performed (see Figure 15). The capacity was slightly decreased from 148 to 142 mAh·g^−1^ when changing cycling rate from C/10 to C/4. After 15 cycles at C/2, the electrode still showed good electrochemical performance with 134 mAh·g^−1^, which delivers over 90% of the capacity at C/10. At the high rate 1C, the electrode maintained a capacity of 117 mAh·g^−1^, which represents approximately 80% of the specific capacity at C/10. After 25 cycles, the current rate was returned to C/10 and the specific capacity was quite similar to the capacity at C/10 for the first ten cycles. Figure 15b shows the rate capabilities of the negative electrode. For the first 10 cycles at C/10, the reversible specific capacity was around 305 mAh·g^−1^. When changing from the rate C/10 to C/4, a stable specific capacity of about 280 mAh·g^−1^ was obtained. A further 10 cycles at C/2 rate yielded a specific capacity of 220 mAh·g^−1^. Even at the high rate of 1C, the electrode kept a specific capacity of above 160 mAh·g^−1^. After 40 cycles at different current rates, the electrode was tested at the rate of C/10 again and achieved a stable specific capacity, which is similar to the first 10 cycles at C/10. The specific capacities at different C rates of the electrodes with lignin as binder were lower than for the electrodes using PVDF as binder. However, these results show that both the positive and negative electrodes with lignin as binder have fairly good rate capabilities. In future work, modifying the lignin as binder will be investigated for higher loadings and better electrochemical properties.

**Figure 16:**
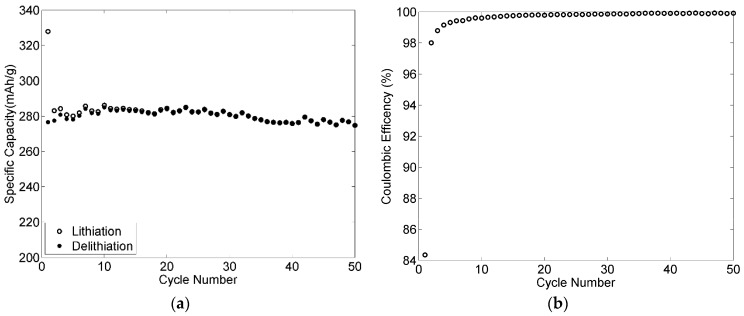
Cycling stability of a 90-2-8 negative electrode cycled at C/4 with 2 wt % VC: (**a**) specific capacity; and (**b**) coulombic efficiency.

**Figure 17:**
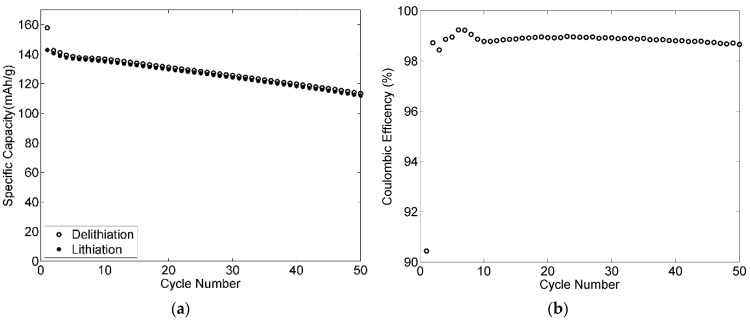
Cycling stability of an 80-11-9 positive electrode cycled at C/3: (**a**) specific capacity; and (**b**) coulombic efficiency.

The cycle performances for both positive and negative electrodes with 50 cycles have been added to the manuscript, as shown in Figure 16 and Figure 17. Very good cycling stability was obtained for the negative electrode as shown in Figure 16. The coulombic efficiency was higher than 99% after three cycles and increased during the experiment to reach 99.9% after 36 cycles. The specific capacity kept relatively stable with about 98% retention after 50 cycles. Figure 17 shows the cycling stability of the positive electrode cycled at C/3. After 50 cycles, the electrode maintained a capacity of 112 mAh·g^−1^, which remains approximately 80% of the initial capacity. This loss is probably caused by the small thickness of the positive electrode, resulting in poor mechanical properties.

## 4. Conclusions

This study is the first attempt to use lignin as binder material for Li-ion battery electrodes, and it shows that lignin is a promising binder material for both LiFeO_4_ positive and graphite negative electrodes. The removal of small dissoluble fractions of the lignin in a pretreatment step is necessary and can be accomplished by leaching in electrolyte. Remaining lignin can be dissolved in acetone for preparation of the electrode slurry together with the other electrode components. Electrodes based on pretreated lignin show relatively high specific capacity and good stability. Pressing of the electrodes did not improve the electrochemical performance. The 80-11-9 electrode was the optimal LiFePO_4_ positive electrode composition with lignin binder yielding the best electrochemical performance. VC addition to the electrolyte can improve the stability of the negative electrodes. Both positive and negative electrodes with lignin binder show good reversible capacity with about 148 mAh·g^−1^ for the positive electrode and 305 mAh·g^−1^ for the negative electrode at C/10. Fairly good rate capabilities can be obtained for the electrodes. At 1C, the specific capacities of the positive and negative electrodes were 117 and 160 mAh·g^−1^, respectively. However, the positive electrodes were thin and further work is needed to modify the lignin in order to produce thicker electrodes with good mechanical properties.

## Figures and Tables

**Table 1:** MMD of the lignin with and without pretreatment.

Sample	Mw (g/mol)	Mn (g/mol)	PD
Original lignin	14,000	1400	9.9
Lignin with pretreatment	16,000	2400	6.8
Extracted fractions	700	400	1.8

**Table 2:** Thermal analysis of lignin with and without pretreatment.

Sample	T_g_ (°C)	T_d_ (5%, °C)	V (250 °C, %)
Original lignin	156	255	95.3
Lignin with pretreatment	146	257	95.5
